# Hospital Statistics: Still More Details Wanted

**Published:** 1887-10-08

**Authors:** Thos. Parkinson

**Affiliations:** Bury, Lancashire


### THE EDITOR'S LETTER-BOX.

*** SEVERAL important letters are unavoidably held over until next week.

HOSPITAL STATISTICS: STILL MORE DETAILS WANTED.

YOUR tables, showing the consumption of milk and alcohol at various hospitals, are very interesting, but would be more instructive if the quantity of milk and the number of resident staff were also given. From a report of the Manchester Infirmary, which I possess, the consumption of milk during the period stated is given at 24,076 gallons, and the total number of patients, officers, and servants resident at 386.82, which gives a daily consumption of .68 of a quart each, which is probably more than the average consumption in hospitals, yet we find that the Newcastle-upon-Tyne Infirmary spent no less than £1,105 11*s*. 6*d*. upon the same article of diet, or £115 more than Manchester, although the total patients at Newcastle only numbered 2,846, against Manchester's 4,455. This shows either that milk is dearer in Newcastle than Manchester, or that the staff, etc., is larger in proportion, and the patients kept in a longer time, or that the consumption is greater by 53 per cent. per head. Other hospitals probably would show as great differences if analysed, and this brings me to the point of the need for a scale of dietary which hospital managers could adopt. If The Hospitals Association Committee could see their way to the recommendation of a suitable scale for ordinary and other diets, I feel sure their efforts would be appreciated by all connected with hospitals.—Yours obediently,

THOS. PARKINSON.

Bury, Lancashire, 29th Sept. 1887.

